# Characteristics and phylogenetic analysis of the complete mitochondrial genome of *Erasmia pulchella* Hope, 1840 (lepidoptera: zygaenidae)

**DOI:** 10.1080/23802359.2024.2317341

**Published:** 2024-02-14

**Authors:** Jihui Zhang, Dandan Zhao, Zhenxing Ma

**Affiliations:** School of Food Science and Biology Engineering, Wuhu Institute of Technology, Wuhu, PR China

**Keywords:** *Erasmia pulchella*, zygaenidae, mitogenome, phylogeny inference

## Abstract

*Erasmia pulchella* has brightly colored wings and releases toxic cyanide as a defense against predation. At present, the molecular phylogenetic status of this species is still unclear. Here, we presented the first complete mitochondrial genome of the genus *Erasmia*, which was assembled from data generated using a genome skimming method. The assembled mitogenome was 15,197 bp in length and consists of 37 genes, including 13 protein-coding genes, two rRNAs, 22 tRNAs, and a control region. Phylogenetic analysis based on both maximum likelihood (ML) and Bayesian inference (BI) revealed that *E. pulchella* was most closely related to *Amesia sanguiflua*.

## Introduction

*Erasmia pulchella* Hope, 1840 is a species in the burnet moth family Zygaenidae. It is widely distributed in Southeast Asia and is the type species of the genus *Erasmia* (Yen et al. 2005a). The larvae of this moth feed on *Helicia cochinchinensis*. The adults generally fly during the day slowly without worrying about being hunted by predators because they are toxic with hydrogen cyanide at all stages of its life cycle (Yen et al. 2005a). When threatened, these toxins foam up from the neck to act as a defense. In addition, their wings are brightly colored, which could be served as aposematic coloration to deter the predators (Yen et al. 2005b).

The genus *Erasmia* formerly contained members of the now-separate genus *Amesia*, which historically occupied a position within *Erasmia* as a subgenus level, but was raised to a genus level later on (Yen et al. 2005b). However, molecular phylogenetic studies of this genus *Erasmia* are still limited. Therefore, to better understand the evolution and phylogeny of Zygaenidae from a molecular perspective, we sequenced the complete mitochondrial genome (mitogenome) of *E. pulchella*, which would facilitate the further understanding of the phylogenetic relationship of both *Erasmia* and Zygaenidae.

## Materials and methods

**Figure 1.**
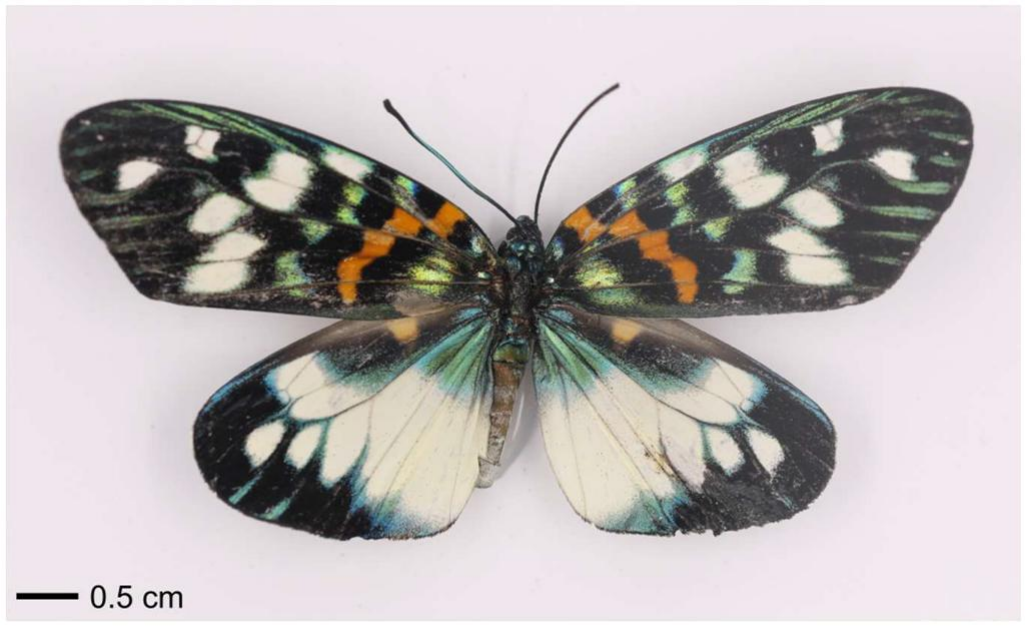
Species reference image of *Erasmia pulchella*. The specimen in this photo was collected in Ji’an City of China (coordinates: E 115.0760, N 27.1165). The image was taken by Jihui Zhang.

Specimen of *E. pulchella* (Figure 1) was collected from Ji’an City of China (E 115.0760, N 27.1165) in July 2021, and identified according to the morphological characters described in Yen et al. (2005b). The muscle was preserved in 95% ethanol and stored at −20 °C. The voucher specimen (number: Lep2102_01) was deposited in Wuhu Institute of Technology in Wuhu city, Anhui Province in China (https://www.whit.edu.cn/index.htm, Jihui Zhang, jihui871031@163.com).

Total genomic DNA was extracted from the thorax of a single specimen with the QIAGEN DNeasy Blood & Tissue Kit (Hilden, Germany) following the manufacturer’s protocols. Genomic libraries were prepared with TruSeq Library Construction Kit with an insert size of 350 bp. The sample was sequenced by Illumina HiSeq 2000 with a paired-end (PE) read length of 150 bp on four lanes. About 8.9 Gb raw data was generated. Fastp v0.20.0 (Chen et al. 2018) was used to remove adaptors and low-quality reads with the parameters “-q 15 -n 10 -u 40.” The mitochondrial genome was assembled using GetOrganelle v1.7.0 (Jin et al. 2020) with default parameters, and annotated by MITOS webserver (http://mitos2.bioinf.uni-leipzig.de/index.py) (Bernt et al. 2013). The depth of coverage was calculated by mapping the reads onto the mitogenome sequence with bowtie2 v2.3.4.3 to determine the correctness of the assembly (Langmead and Salzberg 2012). Finally, the mitogenome was visualized by Chloroplot (Zheng et al. 2020).

Phylogenetic analysis was based on concatenated nucleotide sequences of 13 protein-coding genes derived from nine Zygaenidae species, with three Limacodidae and Tortricidae species as the outgroups. Each nucleotide sequence of the 13 PCGs were translated into amino acids, aligned separately with MUSCLE implemented within MEGA 6.05, and then toggled back into nucleotide alignments. Gblock v0.91b (Castresana 2000) was also employed to eliminate the poorly aligned position and divergent regions. We used IQ-TREE v2.1.3 (Minh et al. 2020) with “-B 1000 -MFP” to construct a maximum likelihood (ML) tree, and Bayesian inference (BI) analysis was estimated using MrBayes 3.2.6 (Ronquist et al. 2012). The final tree was visualized in FigTree v1.4.2 (http://tree.bio.ed.ac.uk/software/figtree/).

## Results

**Figure 2.**
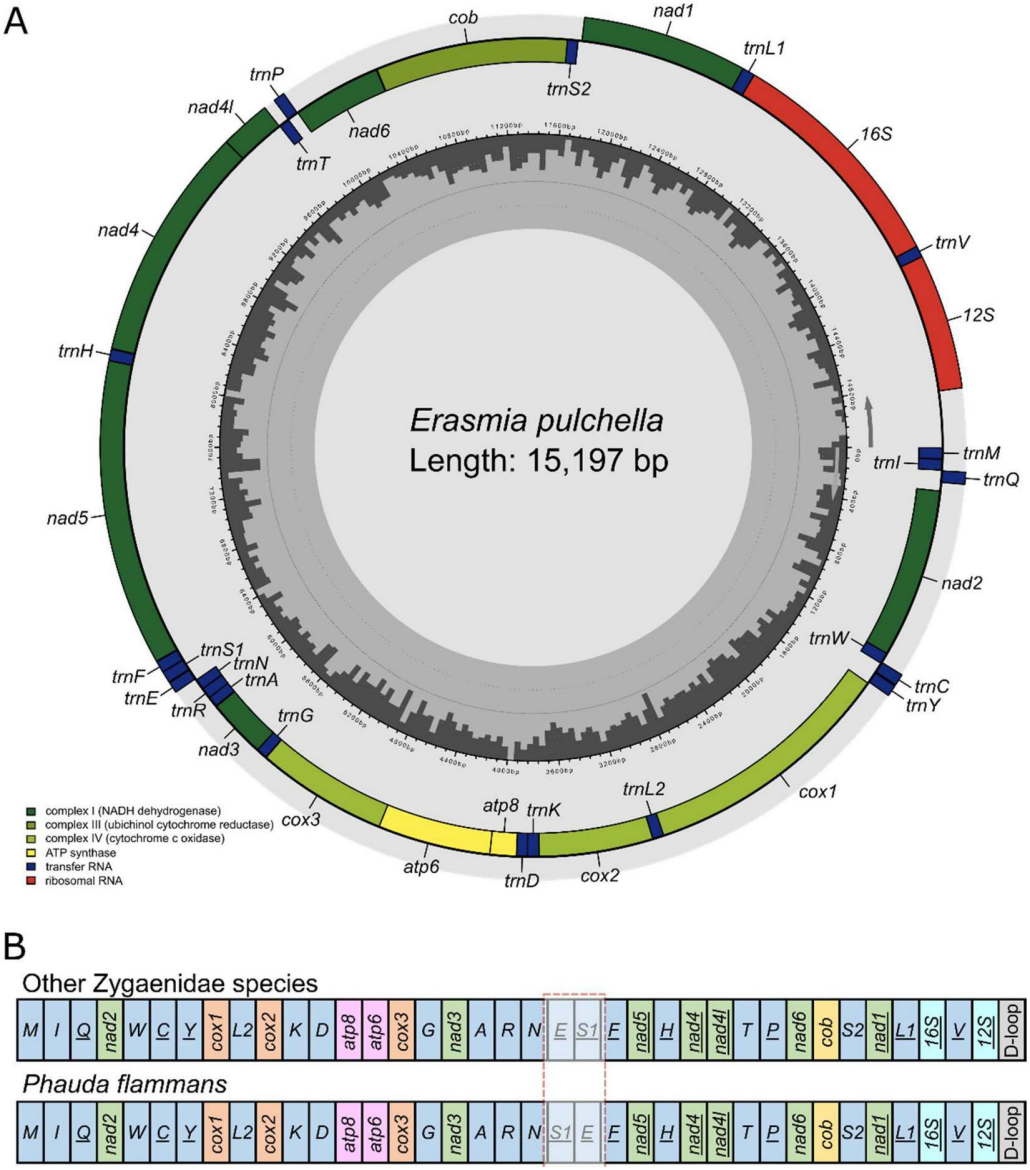
(A) The gene arrangement of the mitochondrial genome of *Erasmia pulchella*. (B) Transposition of *tRNA-Ser* and *tRNA-Glu* in *Phauda flammans* compared with other Zygaenidae species (including *Erasmia pulchella*).

The complete mitogenome of *E. pulchella* was a circular molecule with 15,197 bp in length (GenBank accession number: OQ134124). It contains 13 PCGs, 22 transfer RNAs (tRNAs), two ribosomal RNA (rRNAs) genes, and a non-coding control region (D-loop) (Figure 2(A)). The gene order of the mitogenome of *E. pulchella* is identical with that of other Zygaenidae species, but the transposition of *tRNA-Ser* and *tRNA-Glu* in *Phauda flammans* (Figure 2(B)). All PCGs start with ATN codons, except for cox1 (CGA start codon) and nad1 (GTG start codon). The GTG initiation of *nad1* is also consistent with *Rhodopsona rubiginosa* and *Histia rhodope* in family Zygaenidae (Tang et al. 2014; Peng et al. 2017). 10 PCGs use the standard stop codon TAA, while *nad2*, *cox2*, and *nad4* end with incomplete codon T. The mitogenome of *E. pulchella* was correctly assembled according to the coverage depth (high coverage of over 90 ×) (Figure S1).

**Figure 3.**
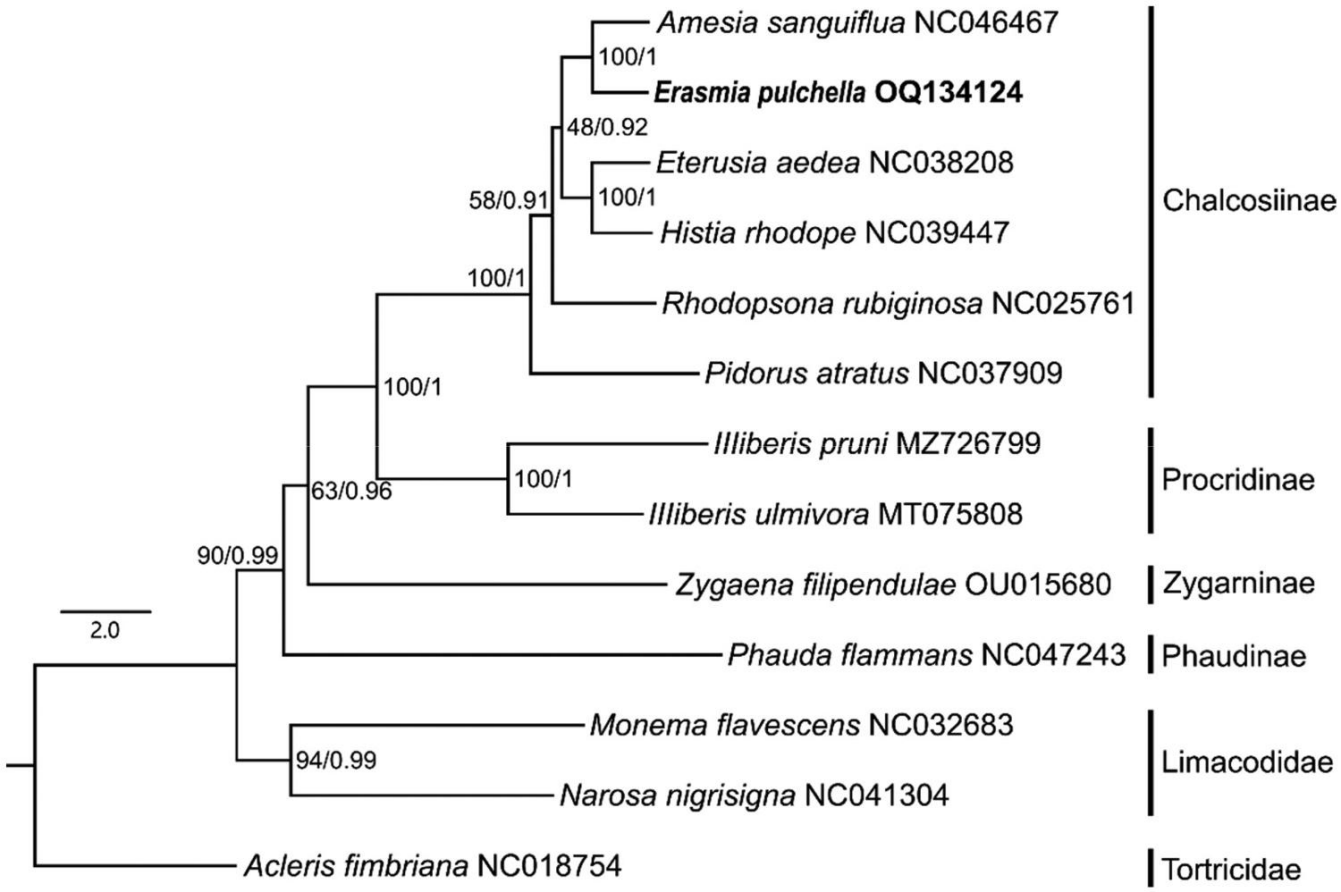
Phylogenetic tree of 10 species for the family Zygaenidae produced from ML analysis based on 13 PCGs dataset. ML and BI support values are shown alongside the nodes, respectively. GenBank accession number is displayed after the species name

The phylogenetic relationships among subfamilies are as follows: (Phaudinae + (Zygaeninae + (Procridinae + Chalcosiinae))) (Figure 3). The newly sequenced species *E. pulchella*, *Eterusia aedea*, *Histia rhodope* and *Amesia sanguiflua* belonging to the subfamily Chalcosiinae were clustered together with high support values (BP = 100; PP = 1).

## Discussion and conclusion

In this study, the mitochondrial genome of *E. pulchella* is reported for the first time. Phylogeny inferred using the ML method based on 13 PCGs showed that Chalcosiinae was the sister lineage of Procridinae. It was also confirmed that *Eterusia* and *Amesia* are sister group, which is consistent with the result based on morphological studies (Yen et al. 2005b). This study not only provides important molecular data for further evolutionary and phylogeographic analysis of *E. pulchella*, but also provides a basis for further study of the phylogenetic relationships within chalcosiine zygaenid moths.

## Supplementary Material

Supplemental Material

## Data Availability

The mitogenome sequence data that support the findings of this study are openly available in GenBank of NCBI at https://www.ncbi.nlm.nih.gov/ under the accession no. OQ134124. Original data was submitted in NCBI, BioProject: PRJNA916127, SRA: SRR22894900, and BioSample: SAMN32413402.
