# Supplementary figures and images for: Characteristics and phylogenetic analysis of the complete mitochondrial genome of Erasmia pulchella Hope, 1840 (lepidoptera: zygaenidae)
Source: Mitochondrial DNA B Resour. 2024 Feb 14;9(2):281–4. doi: 10.1080/23802359.2024.2317341 (PMC10877643; doi:10.1080/23802359.2024.2317341)

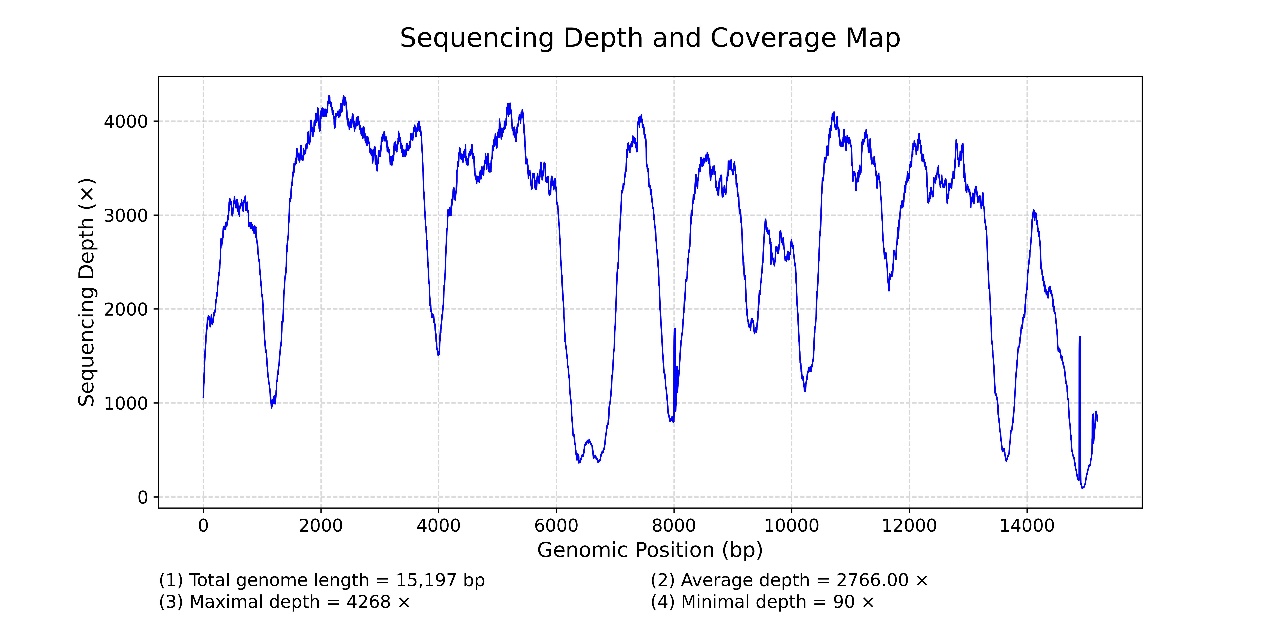


Supplementary Figure 1 The read coverage depth map of *Erasmia pulchella*.

Supplement: Supplemental Material [file TMDN_A_2317341_SM4964.docx]
